# Physical exercise is a risk factor for amyotrophic lateral sclerosis: Convergent evidence from Mendelian randomisation, transcriptomics and risk genotypes

**DOI:** 10.1016/j.ebiom.2021.103397

**Published:** 2021-05-26

**Authors:** Thomas H Julian, Nicholas Glascow, A Dylan Fisher Barry, Tobias Moll, Calum Harvey, Yann C Klimentidis, Michelle Newell, Sai Zhang, Michael P Snyder, Johnathan Cooper-Knock, Pamela J Shaw

**Affiliations:** a Sheffield Institute for Translational Neuroscience (SITraN), University of Sheffield, Sheffield, UK; b Department of Epidemiology and Biostatistics, University of Arizona, Tucson, AZ, USA; c Department of Genetics, Stanford University School of Medicine, Stanford, CA, USA; d Center for Genomics and Personalized Medicine, Stanford University School of Medicine, Stanford, CA, USA

**Keywords:** Amyotrophic lateral sclerosis, Mendelian randomisation, Physical exercise, *C9ORF72*

## Abstract

**Background:**

Amyotrophic lateral sclerosis (ALS) is a universally fatal neurodegenerative disease. ALS is determined by gene-environment interactions and improved understanding of these interactions may lead to effective personalised medicine. The role of physical exercise in the development of ALS is currently controversial.

**Methods:**

First, we dissected the exercise-ALS relationship in a series of two-sample Mendelian randomisation (MR) experiments. Next we tested for enrichment of ALS genetic risk within exercise-associated transcriptome changes. Finally, we applied a validated physical activity questionnaire in a small cohort of genetically selected ALS patients.

**Findings:**

We present MR evidence supporting a causal relationship between genetic liability to frequent and strenuous leisure-time exercise and ALS using a liberal instrument (multiplicative random effects IVW, p=0.01). Transcriptomic analysis revealed that genes with altered expression in response to acute exercise are enriched with known ALS risk genes (permutation test, p=0.013) including *C9ORF72*, and with ALS-associated rare variants of uncertain significance. Questionnaire evidence revealed that age of onset is inversely proportional to historical physical activity for *C9ORF72*-ALS (Cox proportional hazards model, Wald test p=0.007, likelihood ratio test p=0.01, concordance=74%) but not for non*-C9ORF72*-ALS. Variability in average physical activity was lower in *C9ORF72*-ALS compared to both non*-C9ORF72*-ALS (F-test, p=0.002) and neurologically normal controls (F-test, p=0.049) which is consistent with a homogeneous effect of physical activity in all *C9ORF72*-ALS patients.

**Interpretation:**

Our MR approach suggests a positive causal relationship between ALS and physical exercise. Exercise is likely to cause motor neuron injury only in patients with a risk-genotype. Consistent with this we have shown that ALS risk genes are activated in response to exercise. In particular, we propose that G4C2-repeat expansion of *C9ORF72* predisposes to exercise-induced ALS.

**Funding:**

We acknowledge support from the Wellcome Trust (JCK, 216596/Z/19/Z), NIHR (PJS, NF-SI-0617-10077; IS-BRC-1215-20017) and NIH (MPS, CEGS 5P50HG00773504, 1P50HL083800, 1R01HL101388, 1R01-HL122939, S10OD025212, P30DK116074, and UM1HG009442).

**Research in context**

**Evidence before this study**

The role of physical activity as a risk factor for ALS was evaluated in a systematic review of 26 studies performed by Lacorte *et al*. in 2016. The authors concluded that there was insufficient evidence to draw a firm conclusion and highlighted limitations of previous studies relating to heterogeneous classification of both physical activity and ALS. They noted that none of the published literature achieved the highest quality rating in the Newcastle Ottawa Scale, which they attribute to methodological challenges posed by the rarity and severity of the disease. To identify more recent publications, we conducted a literature search using the PubMed database for articles published between 01/01/2015 - 11/11/2020. The search terms used were ("Amyotrophic lateral sclerosis"[Title/Abstract] OR "motor neuron disease"[Title/Abstract] OR MND[Title/Abstract] OR ALS[Title/Abstract]) AND (PA[Title/Abstract] OR exercise[Title/Abstract] OR "physical activity"[Title/Abstract] OR sport[Title/Abstract]). This search strategy yielded 182 results which we filtered for original, observational, human-subject studies; we also excluded case series with <10 participants and case reports. This process identified 12 further relevant publications which reported contrasting conclusions without significantly addressing the methodological issues highlighted above. A single recent study used linkage disequilibrium score regression and Mendelian randomisation to test for a causal relationship between ALS and a number of UK biobank questionnaire items including participation in light DIY, walking for pleasure and moderate activity duration, but this study did not address the relationship between ALS and physical exercise which is both frequent and strenuous. This study concluded that there was a correlation between genetic liability to physical exercise and ALS but they did not record a positive MR result to indicate causation.

**Added value of this study**

In the present study, we have exploited the methodological advantages of Mendelian randomisation (MR) to counter bias, together with a tailored approach to physical activity exposure aimed at isolating exercise which is both frequent and strenuous. We achieved this by selecting and combining UK biobank questionnaire items. We present the first MR evidence of physical exercise as a causative factor in the development of ALS. Furthermore, we have addressed the gene-environment interaction by measuring the effect of exercise on expression of ALS risk genes. Finally, we have considered in detail the relationship between physical activity and the most frequent genetic risk factor for ALS: hexanucleotide (G4C2) repeat expansion of *C9ORF72*. Our data suggest that genetic liability to leisure time physical activity is a risk factor for ALS and *C9ORF72*-ALS in particular. In addition, we offer evidence that a number of known ALS-associated genetic variants are functionally linked to the physiological response to exercise.

**Implications of all the available evidence**

Our results indicate that participation in frequent and strenuous leisure time physical activity is a risk factor for ALS, particularly in the context of certain risk genotypes. This could explain some of the controversy in previous studies which have largely neglected genetic heterogeneity within ALS patients. Our results form a platform for future research to explore the interaction between specific genotypes and exercise-induced ALS in a prospective manner with larger numbers, and in selected pedigrees. Ultimately this could lead to the design of a personalised medicine approach including lifestyle advice regarding physical activity, to patients with ALS and their family members.

Alt-text: Unlabelled box

## 1 Introduction

Amyotrophic lateral sclerosis (ALS) is a devastating, rapidly progressive and relatively common neurodegenerative disease with a lifetime risk of ~1/400 [1]. ALS is thought to result from interplay between the environment and risk-genotypes [2]. Like other complex diseases, the risk of ALS has a significant heritable component [3,4], but the penetrance of specific genetic variants is variable which is consistent with environmental modifiers. ALS is notable for the late age of onset, even in rare monogenic forms, and this has been interpreted as a ‘multiple-hit’ process involving sequential genetic and environmental insults [5,6]. Identification of specific gene-environment interactions may open the door to personalised medicine and even disease prevention.

To date, identification of environmental risk factors for ALS has been limited. ALS has a higher incidence and lower age of onset in professional sportspeople which led to the proposal that exercise is a risk factor for ALS [7]. However, epidemiological studies attempting to quantify exercise-history in ALS patients have produced conflicting results [8], [9], [10], [11]. These studies have largely relied on questionnaire-based quantification of exercise in an unselected cohort of ALS patients. Inherent in these approaches is selection bias, recall bias, and confounding due to the effect of exercise on other causes of mortality. In particular, failure to consider genetic heterogeneity within ALS cohorts may have masked specific gene-environment interactions. Of the conflicting results that have been published, it is potentially significant that a positive relationship between ALS risk and exercise has often been reported in populations with high incidence of the G4C2 repeat expansion within *C9ORF72* [9,12,13]. *C9ORF72* expansion is the most common genetic risk factor for ALS, but shows marked phenotypic variability including incomplete penetrance [14]. This variability has led to the proposal of various genetic and environmental modifiers, but to date no specific modifier has been conclusively demonstrated.

Exercise itself is a heterogeneous activity. Muscle fibres and motor neurons are sub-specialised for aerobic and anaerobic conditions. Skeletal muscle fibres are categorised as fast twitch (type IIa, IIb and IIx) or slow-twitch (type I) according to functional (e.g. contractile speed) and metabolic properties [15]. In ALS it is the motor neurons supplying type IIb muscle fibres responsible for anaerobic burst activity which are most vulnerable to the disease process [16,17]. On this basis we propose that ALS may be associated specifically with vigorous exercise and indeed this hypothesis is consistent with several previous studies [9,18]. In designing the present study we therefore focused on frequent and intense, anaerobic, burst activity often undertaken in leisure, training and competitive physical exercise activities [19].

**Fig. 1:**
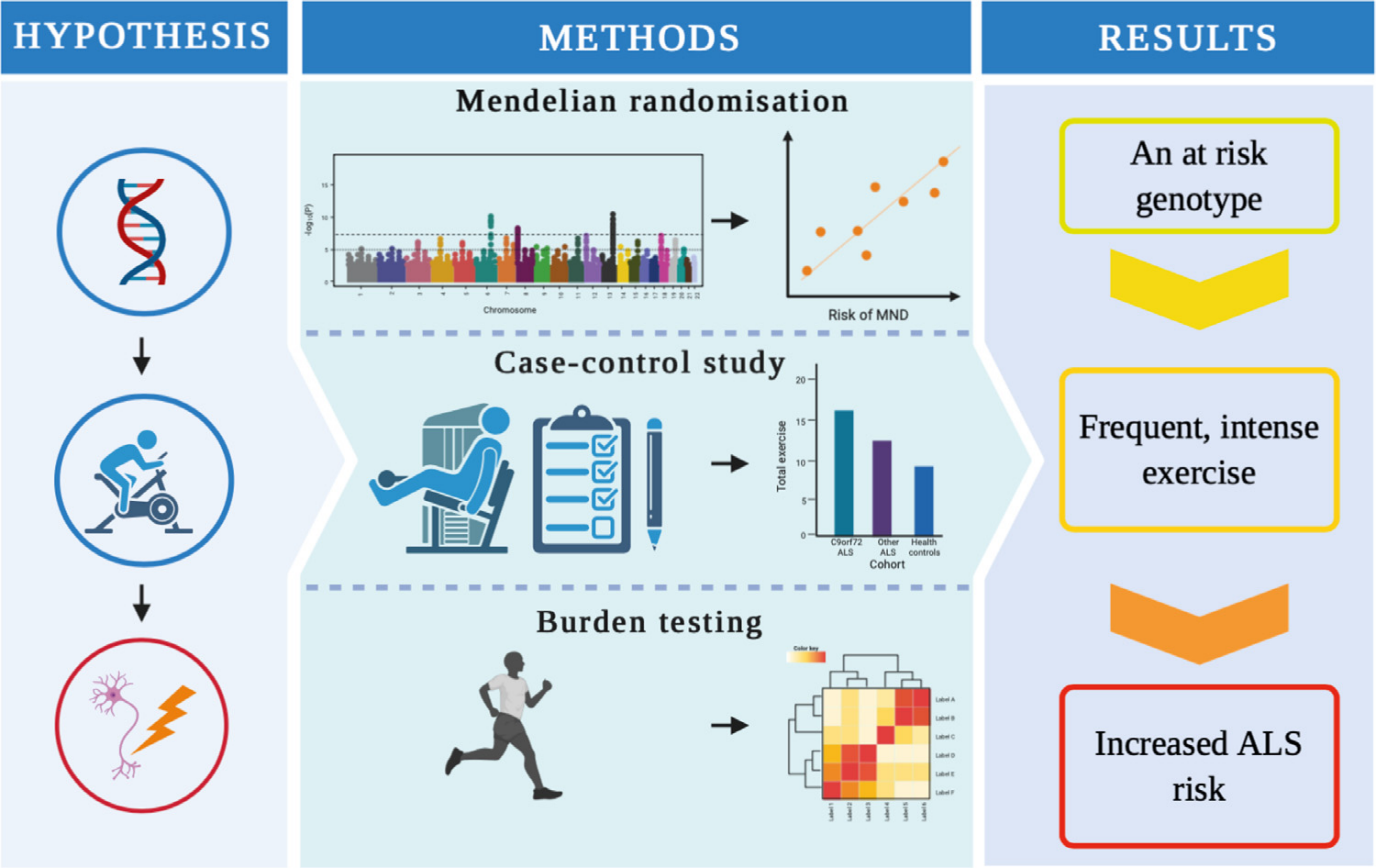
Exercise is a risk factor for amyotrophic lateral sclerosis: Convergent evidence from Mendelian randomisation, transcriptomics and risk genotypes. A graphical abstract describing the fundamental elements of the study and core results. ALS = amyotrophic lateral sclerosis; PA = physical activity; SNP = single nucleotide polymorphism; GWAS = genome wide association study.

Two-sample Mendelian randomisation (MR) allows evaluation of the relationship between an exposure and an outcome through upstream genetic correlates in independent cohorts. Genetic correlates are measured in a genome-wide association study (GWAS). This ameliorates much of the bias which has confounded previous studies of historical-exercise in ALS patients. We set out to use MR to test whether physical exercise is a risk factor for the development of ALS. We hypothesised that some of the inconsistency between previous studies may reflect heterogeneity in exercise measures and we therefore focused on GWAS measuring frequent and strenuous leisure-time activity. To address genetic heterogeneity we evaluated the association between exercise-associated gene expression changes and ALS risk, and we have evaluated historical physical activity in a small cohort of genetically homogeneous ALS patients. Our study approach is summarised in Fig. 1.

## 2 Methods

### 2.1 Two-sample Mendelian randomisation (MR)

#### 2.1.1 Objective

In MR, SNPs associated with an exposure of interest are used as instrumental variables (IV) to explore the causal relationship between that exposure and an outcome of interest. According to Mendel's laws of segregation and independent assortment, this methodology can be considered a natural experiment in which individuals are at conception randomly assigned to groups with differing levels of genetic liability to an exposure of interest. A core strength of MR is the ability to remove unmeasured confounding and to differentiate between a shared genetic basis for the exposure and outcome (known as genetic pleiotropy), and potential causation. By utilising SNPs associated with various forms of exercise, we are therefore able to interrogate the relationship between exercise and ALS. SNPs which make up the IV are usually selected on the basis of genome wide association studies (GWAS) used to assess the significance of the relationship between specific genetic variants and an exposure of interest [20].

#### 2.1.2 Study participants

In this study, the instrumental variables (IV) were derived from publicly available GWAS data. None of the exposure GWAS participants overlapped with those in the ALS outcome GWAS which is important to avoid bias.

SSOE was measured via questionnaire in UK Biobank (UKB) participants [21]. Individuals completing the physical activity questionnaire were also asked “In the last 4 weeks did you spend any time doing the following?” with the potential answers were: ‘walking for pleasure’, ‘other exercises’, ‘strenuous sports’, ‘light DIY’, ‘heavy DIY’, ‘none of the above’, and ‘prefer not to answer’. To address the question of which SNPs were associated with frequent participation in “strenuous sports” and “other exercise” (SSOE) the responses were dichotomised to compare those who reported spending two-three days per week or more performing SSOE for a duration of 15-30 minutes or greater, with those and who did not report spending any time within the last four weeks performing SSOE. A total of 124,842 cases and 225,650 controls contributed to the SSOE GWAS. The data were analysed with a linear mixed model. The UKB data field references for the questions used to create this GWAS are 6164, 3647, 1001, 3637 and 991.

We carried out a number of sensitivity analyses to check whether association with ALS was specific to frequency and strenuous physical exercise. We evaluated the relationship between ALS and participation in strenuous sport during the last 4 weeks with no minimum threshold for frequency or duration [22]; and heavy DIY which was excluded from the SSOE measure [22]. These GWAS also utilised the UKB questionnaire; they included 460,376 subjects and analysis was performed using a linear mixed model. To quantify movement independent of exercise we utilised accelerometer data: UKB subjects wore an Axivity AX3 accelerometer for one week, a linear mixed model GWAS analysis was conducted on the basis of three measures: 1) overall average acceleration; 2) fraction of accelerations > 425 milligravities; 3) sedentary behaviour [21,23]. 91,084 individuals data contributed to the average accelerations analysis, 90,667 to the GWAS of fraction of accelerations >425 milligravities and 91,105 to the GWAS of sedentary behaviour [23].

Body fat was measured by dual energy x-ray absorptiometry or bioimpedance analysis [24]. Body fat GWAS was performed as a meta-analysis and included 100,716 individuals.

Educational attainment was quantified as years of education measured at an age of at least 30 years; GWAS was performed in a meta-analysis of 1,131,881 individuals [25].

ALS GWAS was performed utilising a linear mixed model as previously described [26]. This is the largest published ALS GWAS to date where a linear mixed model (LMM) was used to correct for population differences [27]; this GWAS included 12,577 cases and 23,475 controls from 41 cohorts. It has been previously demonstrated that a LMM can achieve improved power over a meta-analysis without significant false positives [26].

Limitations of GWAS datasets are discussed in the **Supplementary Note**.

#### 2.1.3 Selection of exposure instrumental variables (IV)

Exposure IV are chosen based on an arbitrary p-value cut off [28,29]. A cut-off which is too low will lose informative instruments, but a cut-off which is too high could introduce non-informative instruments. We chose to utilise a positive control to determine the optimum number of IV to measure SSOE; this is independently supported best practice [20]. Body fat percentage was chosen as a positive control because there is a clear and described biological mechanism linking increased exercise to reduced body fat. We compared a liberal (p<1E-06) and conservative (p<5E-08) instrument to test the relationship between SSOE and body fat percentage; both results were significant but the liberal instrument had a greater power. Moreover, certain of the robust analyses using the conservative instrument paradoxically suggested that increased physical exercise should increase body fat percentage (Table 2**, Supplementary Table 8**). We note that others have concluded that a conservative instrument is an underpowered measure of SSOE [28]. Consequently, we have used a liberal instrument to measure SSOE in our analysis.

Identified SNPs at each significance threshold were clumped for independence using PLINK clumping in the TwoSampleMR tool [30]. A stringent cut-off of R^2^ ≤ 0.001 and a window of 10,000kb was used for clumping within a European reference panel. Where SNPs were in linkage disequilibrium (LD), those with the lowest p-value were retained. SNPs which were not present in the reference panel were excluded. Where an exposure SNP was unavailable in the outcome dataset, a proxy with a high degree of LD (R^2^ ≥ 0.9) was identified in LDLink within a European reference population [31]. Where a proxy was identified to be present in both datasets, the target SNP was replaced with the proxy in both exposure and outcome datasets in order to avoid phasing issues [32]. Where a SNP was not present in both datasets and no SNP was available in sufficient LD, the SNP was excluded from the analysis. All SNPs selected for inclusion in this study are presented in the supplementary data in order to allow replication.

#### 2.1.4 Exposure-outcome instrument harmonisation

The effects of SNPs on outcomes and exposures were harmonised in order to ensure that the beta values were signed with respect to the same alleles. For palindromic alleles, those with minor allele frequency (MAF) > 0.42 were omitted from the analysis in order to reduce the risk of errors due to strand issues [32].

#### 2.1.5 Assumptions and robust analyses

The MR measure with the greatest power is the inverse-variance weighted (IVW) method, but this is contingent upon the exposure IV assumptions being satisfied [33]. With the inclusion of a large number of SNPs within the exposure IV, it is possible that not all variants included are valid instruments and therefore, in the event of a significant result, it is necessary to include a range of robust methods which provide valid results under various violations of MR principles at the expense of power [20]. Robust methods applied in this study include MR-Egger, MR-PRESSO, weighted median, weighted mode and the robust adjusted profile score (RAPS).

With respect to the IVW analysis, a fixed-effects (FE) model is indicated in the case of homogeneous data, whilst a multiplicative random effects (MRE) model is more suitable for heterogeneous data. Burgess *et al* recommend that an MRE model is implemented when using GWAS summary data to account for heterogeneity in variant-specific causal estimates [20]. In the interest of transparency, we calculated both results but present the MRE in the text.

MR analyses should include evaluation of exposure IV strength. In order to achieve this, we provide the F-statistic, MR-Egger intercept, MR-PRESSO global test, Cochran's Q test and I^2^ for our data. The F-statistic is a measure of instrument strength with >10 indicating a sufficiently strong instrument [34]. We provide F-statistics for individual exposure SNPs and the instrument as a whole. Cochran's Q test is an indicator of heterogeneity in the exposure dataset and serves as a useful indicator that horizontal pleiotropy is present as well as directing decisions to implement FE or MRE IVW approaches [35]. The MR-Egger intercept test determines whether there is directional horizontal pleiotropy. The MR-PRESSO global test determines if there are statistically significant outliers within the exposure-outcome analysis [36]. I^2^ was calculated as a measure of heterogeneity between variant specific causal estimates, with a low I^2^ indicating that Egger is more likely to be biased towards the null [37]. Finally, we performed a leave-one-out analysis using the method of best fit for each exposure SNP within the IV in order to determine if any single variants were exerting a disproportionate effect upon the results of our analysis [20].

### 2.2 Linkage disequilibrium score regression (LDSC)

#### 2.2.1 Objective

LDSC is a tool which can be utilised in order to evaluate genetic correlation between traits using GWAS summary statistics. This method does not however distinguish between genetic correlation (pleiotropy) and causation.

#### 2.2.2 Study participants

The aforementioned SSOE and ALS GWAS data were utilised in LDSC.

#### 2.2.3 Analysis

For the most part, we did not deviate from the default settings recommended by authors of the LDSC package [38,39]. SNPs were filtered according to presence in HapMap3, MAF > 0.01, removal of strand ambiguous SNPs and duplicated SNPs. In our analysis we offer both a regression estimate without constraining the heritability estimates of ALS and SSOE and with a constrained ALS intercept as recommended previously [40].

### 2.3 Burden testing

Whilst GWAS is useful for exploring the genetic architecture of disease, this methodology does not capture low-frequency or rare variation [41] which is key to the genetic architecture of ALS [26]. Rare variants are addressed by rare variant burden testing [42]. In this study, we grouped genes within pathways which changed expression in response to exercise. We measured the rate of observed rare mutations within exercise pathways in ALS patients compared to controls. By identifying ALS-associated genetic variation within pathways functionally related to acute exercise, we aimed to implicate specific genotypes in motor neuron vulnerability to PA.

Burden testing was performed using whole genome sequencing data from 4,425 ALS patients and 1,925 controls [43]. We have previously identified genes and pathways differentially expressed in response to exercise [44]. In brief 36 subjects underwent symptom-limited cardiopulmonary exercise (CPX) testing with whole transcriptome profiling by sequencing of RNA extracted from peripheral blood mononuclear cells at 2, 15, 30 and 60 minutes post exercise [44]. For the purpose of the present research, pathways which were differentially expressed at the most immediate time point of 2 minutes post-CPX testing (p<0.001) were selected for burden testing. For each pathway we calculated the proportion of genes which were significantly enriched with rare (MAF<1%) ALS-associated mutations which alter amino-acid sequence [43]. To determine significant enrichment of each pathway with ALS-associated mutations we calculated the equivalent enrichment within 5,000-30,000 random gene sets of equivalent length. Reported p- and FDR values for pathway enrichment refer to the proportion of random gene sets with equivalent enrichment. To ensure stable p-value estimates the number of random gene sets was increased such that ≥3 random sets were discovered with equivalent enrichment to each pathway under consideration.

### 2.4 Expression of ALS-related genes during exercise

In order to determine whether ALS-related genes are differentially expressed during physical exercise, we measured expression changes of known ALS genes post-exercise [44]. We utilised a clinical ALS gene panel (Next Generation Sequencing at Sheffield NHS Children's Hospital, **Supplementary Table 9**) [45]. The proportion of ALS genes which were differentially expressed with exercise was compared to a 1,000 random gene sets of the same length.

### 2.5 A *C9ORF72*-specific case-control study of historical PA

#### 2.5.1 Study participants

ALS cases were identified by a Consultant Neurologist according to revised El-Escorial criteria. Cases with family history or other clinical features consistent with *C9ORF72*-disease such as young age of onset or extra-motor features underwent genetic testing for the G4C2-expansion. Inclusion criteria for the study were as follows: (1) Confirmed *C9ORF72* pathological G4C2-repeat expansion; (2) patients were over 18 years of age whose disease manifested in adult life; and (3) diagnosis had taken place within the last two years. Exclusion criteria were as follows: (1) patients with concurrent neurological disease; (2) patients with symptoms incompatible with completing the HAPAQ questionnaire such as overt cognitive impairment. Participation was maximised by travelling to administer the questionnaire in the patient's place of residence; in this way we managed to avoid any failed recruitment and consequent selection bias.

Two control groups were used in this study: those with ALS but without the *C9ORF72* G4C2-expansion, and neurologically normal controls. Both groups were matched to *C9ORF72*-ALS patients for age and gender. Control data were obtained from a previous study [9]. Participants in the study are summarised in Table 4.

#### 2.5.2 Measurement of physical activity

Physical activity was measured using the HAPAQ questionnaire which has been previously validated for the determination of historical PA [46]. Briefly a life calendar was used to orientate participants and aid recall. The questionnaire is structured by time period, of which two broad lengths of time are covered (a) the most recent 15 years, split into three 5-year categories and (b) the whole of adulthood, by decade, starting from the age of 20 years. In each discrete time period a series of questions are asked about physical activity within four distinct domains: (a) in and around the home; (b) at work or commuting to work; (c) physical activity that makes you out of breath/sweat and (d) physical activity that does not make you out of breath or sweat. For each domain, closed questions are asked about the type, duration, and frequency of the PA.

#### 2.5.3 Statistical analysis

Every unique activity was assigned a MET value according to the Compendium of Physical Activities [47]. Based on these values we calculated a measure of the average daily physical activity in kJ/kg/day for each patient during each time period which accounted for type, duration, intensity, and frequency of PA. To compare between subjects an overall average daily physical activity value was calculated for the most recent 20 years excluding the most recent 5 years which may have been confounded by subclinical symptom onset.

### 2.6 Software

The TwoSampleMR (version 0.5.5) package in R (version 4.0.2) was used to perform Mendelian randomisation [48]. Proxy SNPs for Mendelian randomisation were identified using the LDLinkR (version 1.0.2) package [49]. The code which we utilised for the more statistically complex aspects of the work (MR and burden analysis) is provided in the **Supplementary Note.** R language was also utilised for burden testing and various basic calculations, performed using base R and dplyr functionality. The statistics program STATA/IC (version 15.0) was used for statistical analysis of case-control data. The LDSC (version 1.0.1) package was operated using Anaconda Python (version 3.8) in order to produce LD score regression estimates.

### 2.7 Ethics

Relevant National Health Service (NHS) and university research ethical approvals were obtained (IRAS189432 / STH19120) and procedures followed were in accordance with these standards. All study participants provided informed consent.

### 2.8 Role of funding source

Funders had no role in study design, data collection, data analyses, interpretation, or writing of the manuscript.

## 3 Results

### 3.1 Genetic liability to frequent, strenuous, leisure-time exercise is a risk factor for ALS

We hypothesised that genetic liability to exercise is associated with ALS. Exercise is not a single exposure, but is heterogeneous both in terms of the activity performed and the metabolic, neuromuscular and cardiovascular consequences. Our previous work has indicated that leisure-time strenuous activity may be linked to risk of ALS [9]; this is captured by a ‘strenuous sport and other exercise’ (SSOE) GWAS (**Methods**) [21]. Importantly this measure includes thresholds for both intensity and frequency of physical activity: 2-3 days per week or more performing SSOE for a duration of 15-30 minutes or greater. Interestingly, occupational physical activity is negatively associated with the SSOE measure, suggesting that SSOE is a relatively specific measure of frequent and intense leisure-time activity [21]. As controls we have employed GWAS of (i) strenuous sport with a lower frequency threshold: performed at any time during the last 4 weeks; (ii) heavy DIY; and (iii) an accelerometer study of total movement over a limited period (**Methods**).

**Fig. 2:**
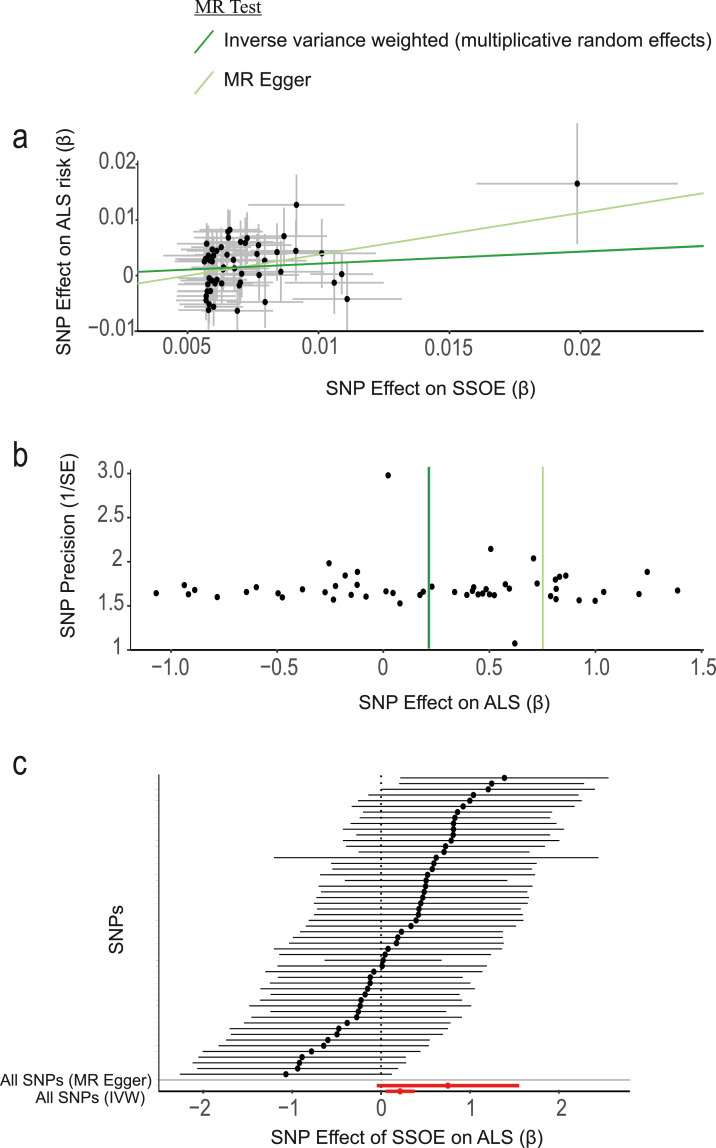
Genetic liability to frequent, strenuous, leisure-time exercise is a risk factor for ALS. **(a)** Scatter plot demonstrating the positive correlation between genetic liability to strenuous sport and other exercise (SSOE) and ALS, as measured with a liberal instrument. Points indicate effect size (β) and standard errors for each SNP-outcome relationship (i.e. ALS and SSOE). The relationship is not significantly altered by removal of any single SNP. The weighted median estimator is not significantly different to the IVW (β = 0.22 and 0.21 respectively) and therefore cannot be independently visualised. **(b)** A symmetrical funnel plot (vertical line of best fit) demonstrates that SNP effect size (β) is not correlated with SNP precision i.e. inaccurate instruments are not overvalued which could lead to directional pleiotropy. (**c)** Forest plot illustrates that the effect of SSOE on ALS is consistent whether measured by individual SNPs or by MR Egger (upper red line) or IVW (lower red line). The overlapping confidence intervals of each causal estimate show there is no significant heterogeneity.

We discovered that genetic liability to SSOE is positively associated with ALS using a liberal instrument (IVW p=0.01, beta=0.21, Table 1**,** Fig. 2**, Supplementary Table 1**). Use of a liberal instrument is supported by analysis of a positive control where we compared a liberal and a conservative instrument to measure the effect of SSOE on body fat percentage (**Methods,** Table 2). There was no statistically significant heterogeneity or directional pleiotropy and the F-statistic indicates adequate instrument strength (Table 3). MR-PRESSO global test and leave-one-out analysis did not demonstrate any SNP outliers (Table 3). Robust tests consistently reached or were close to significance (Table 1). This result is consistent with a causative relationship between frequent and strenuous exercise, and ALS which is not confounded by selection or recall bias. This result suggests that the association is driven primarily by a deleterious effect of exercise on motor neuron health and not by horizontal pleiotropy, i.e. our data are not consistent with a common genotype which independently influences both exercise and ALS (**Methods**).

**Table 1:** Two-sample Mendelian randomisation demonstrates that strenuous sport and other exercise (SSOE) is a risk factor for ALS using a liberal instrument.

Instrument	Mendelian randomisation method	Beta	Standard error	p value
**Liberal (p<1E-06)**	Inverse variance weighted (fixed effects)	0.21	0.08	0.007
	Inverse variance weighted (multiplicative random effects)	0.21	0.08	0.01
	MR Egger	0.75	0.41	0.07
	Weighted median	0.22	0.11	0.05
	Weighted mode	0.52	0.25	0.04
	MR RAPS with overdispersion model	0.21	0.09	0.009
	MR RAPS without overdispersion model	0.24	0.08	0.005
**Conservative (p<5E-08)**	Inverse variance weighted (fixed effects)	0.28	0.16	0.07
	Inverse variance weighted (multiplicative random effects)	0.28	0.18	0.11
	MR Egger	-1.09	0.89	0.26
	Weighted median	0.17	0.22	0.44

**Table 2:** Positive control two-sample Mendelian randomisation analysis of the relationship between strenuous sport and other exercise (SSOE), and body fat percentage. This analysis was used to chose the best instrument for analysis of the relationship between SSOE and ALS. The liberal instrument appears to produce more precise estimates with smaller standard errors and greater power, due to the larger number of SNPs. Although MR RAPS is significant in both the liberal and conservative analysis, the conservative tool paradoxically identifies a positive relationship between SSOE and body fat percentage.

Positive control instrument	Multiplicative random effects IVW beta	Fixed effects IVW p-value	Multiplicative random effects IVW p-value	Weighted median p-value	MR-Egger p-value	Weighted mode p-value	MR RAPS p-value
**Conservative (p<5E-08)**	-0.78	0.003	0.01	0.23	0.97	0.37 (falsely positive beta)	0.04 (falsely positive beta)
**Liberal (p<1E-06)**	-0.64	0.000004	0.00002	0.03	0.85	0.64	0.0004 (correctly identified negative beta)

**Table 3:** Two-sample Mendelian randomisation for the effect of strenuous sport and other exercise (SSOE) on risk of ALS consists of robust instrumental variables.

Statistical test	Salient results
Cochran's Q (for IVW)	p=0.31
MR-Egger intercept	p=0.18
Leave-one-out with fixed effects IVW	P=0.003-0.02 with a consistently positive beta.
I^2^ test	0.96
F statistic for the combined instrument	28.15
MR-PRESSO global test	p=0.33

Movement alone was not significantly associated with ALS, which is consistent with our hypothesis that ALS is linked to strenuous exercise. MR determined a non-significant association with ALS for fraction of accelerations >425 milligravities (IVW, p=0.13, beta=-0.11, **Supplementary Table 2, Supplementary Fig. 1**) and average accelerations (IVW, p=0.31, beta=-0.005, **Supplementary Table 3, Supplementary Figure 2**). Moreover, neither infrequent ‘strenuous sport’ (IVW, p=0.13, beta=0.35, **Supplementary Table 4, Supplementary Figure 3**) or heavy DIY (IVW p=0.17, beta=-0.17, **Supplementary Table 5, Supplementary Figure 4**) were significantly associated with ALS. Overall, our data are consistent with the hypothesis that risk of ALS is linked to frequent and intense leisure-time physical activity.

Linkage disequilibrium score regression (LDSC) is a technique to determine whether two traits share a common genetic architecture. Unlike MR, LDSC cannot distinguish a causal relationship between exposure and outcome from pleiotropy. Consistent with our MR result, a LDSC analysis revealed a positive genetic correlation between ALS and SSOE (r=0.17, standard error=0.09, **Supplementary Table 6**) which was borderline statistically significant (p=0.06). In a previous analysis using the same ALS GWAS data, the authors constrained the intercept for heritability of ALS to 1 on the basis that population structure and confounding were carefully controlled in the ALS GWAS [40]. If we employ the same strategy then the correlation between SSOE and ALS becomes statistically significant (r=0.08, standard error=0.04, p=0.04, **Supplementary Table 6**).

### 3.2 Sedentary behaviour is not significantly associated with ALS

We argued that if strenuous exercise is a risk factor for ALS, then sedentary behaviour may be protective. However, our MR study does not support this conclusion. Sedentary behaviour is not significantly related to ALS (IVW p=0.29, beta=-0.04, **Supplementary Table 7, Supplementary Figure 5**).

### 3.3 Association between physical exercise and ALS is not mediated by body fat percentage

Body fat percentage has previously been described as a potential risk factor for ALS [50]. If true this would represent a possible source of pleiotropy or confounding in the SSOE-ALS relationship. However, our analysis demonstrates that body fat percentage is not significantly associated with ALS (IVW, p=0.43, beta=-0.03, **Supplementary Table 9, Supplementary Figure 6**) and is therefore unrelated to the pathophysiological impact of SSOE. This finding is in keeping with a previous MR which concluded that there is no causal relationship between BMI and ALS [51].

### 3.4 Association between exercise and ALS is not mediated by educational attainment

The relationship between years of education and ALS was measured to identify whether this variable might have been a confounder for the SSOE-ALS relationship. Previous studies have demonstrated that leisure-time SSOE is positively correlated with educational attainment suggesting that our result could be confounded by a link between lower educational attainment and increased risk of ALS [21]. Due to the large number of SNPs identified at genome-wide significance which were eligible for analysis (n=298), only a conservative instrument was used. This demonstrated no significant relationship between ALS and educational attainment (IVW p=0.64, beta=-0.01, **Supplementary Table 10, Supplementary Figure 7**).

### 3.5 Exercise-induced pathways are enriched with ALS genetic risk

**Fig. 3:**
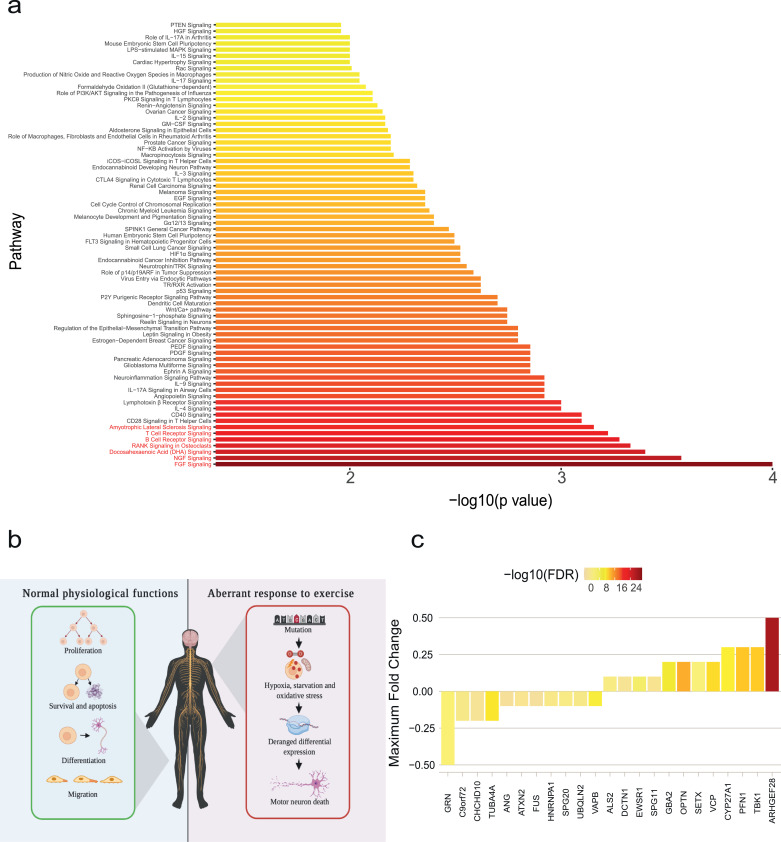
Exercise-induced pathways are enriched with ALS genetic risk. **(a)** Transcriptome analysis of peripheral blood mononuclear cells (PBMCs) reveals that biological pathways differentially expressed following acute exercise are significantly enriched with ALS-associated rare variants. The pathways depicted pass multiple testing correction (FDR<0.05). **(b)** Differentially expressed biological pathways are closely related to neuronal health. Broadly, the roles of fibroblast growth factor (FGF) and nerve growth factor (NGF) pathways are cell proliferation, apoptosis and cell survival, cell differentiation and cell migration. The NGF and FGF pathways are significantly related to ALS and are enriched following acute exercise. In ALS, it is possible that mutation in these pathways leads to deranged differential expression in response to hypoxia, oxidative stress and starvation during exercise and this may precipitate damage to motor neurons. **(c)** ALS risk genes which are differentially expressed in response to exercise are shown with fold change and significance.

Our results suggest that physical exercise is a risk factor for ALS. We set out to identify risk genotypes for exercise-induced ALS using transcriptomics. We hypothesised that risk genes for exercised-induced ALS should be differentially expressed in response to exercise. This could provide a functional link whereby exercise amplifies toxicity resulting from a genetic mutation. We measured gene expression changes in peripheral blood mononuclear cells (PBMCs) in response to exercise and identified 323 biological pathways that are differentially expressed in response to acute exercise including ‘ALS signaling’ [44]. We tested for enrichment of ALS genetic risk within each of these pathways by rare variant burden testing utilising whole-genome sequencing (WGS) data from 4,495 sporadic ALS patients and 1,925 controls (**Methods** and [43]). Our focus on rare variants was guided by previous work demonstrating that ALS has a polygenic rare variant architecture [26]. Strikingly 72 pathways (22%) are significantly enriched with ALS-associated rare deleterious variants after multiple testing correction (FDR<0.05, Fig. 3**a, Supplementary table 11**) including the ‘ALS signaling’ pathway (p=0.0007). One pathway was significantly enriched with ALS-associated rare variants even after a stringent Bonferroni multiple testing correction: fibroblast growth factor (FGF) signaling (p=0.0001, Fig. 3a-b). Nerve growth factor (NGF) signaling is closely related to FGF signaling and is also highly enriched with ALS-associated rare variants (p=0.0002, Fig. 3a-b).

We hypothesised that, if exercise-induced gene expression changes are enriched with ALS risk, then known ALS genes should also be differentially expressed following exercise [44]. Consistent with our hypothesis 52% of clinically validated ALS-related genes are differentially expressed following acute exercise and this enrichment is statistically significant (permutation test, p=0.013, Fig. 3**c, Supplementary Table 12, Methods**). The list of differentially expressed genes included down-regulation of *C9ORF72* (fold change=-0.2, FDR=0.0002).

### 3.6 Case-control study of *C9ORF72*-ALS suggests that exercise is a disease modifier

**Fig. 4:**
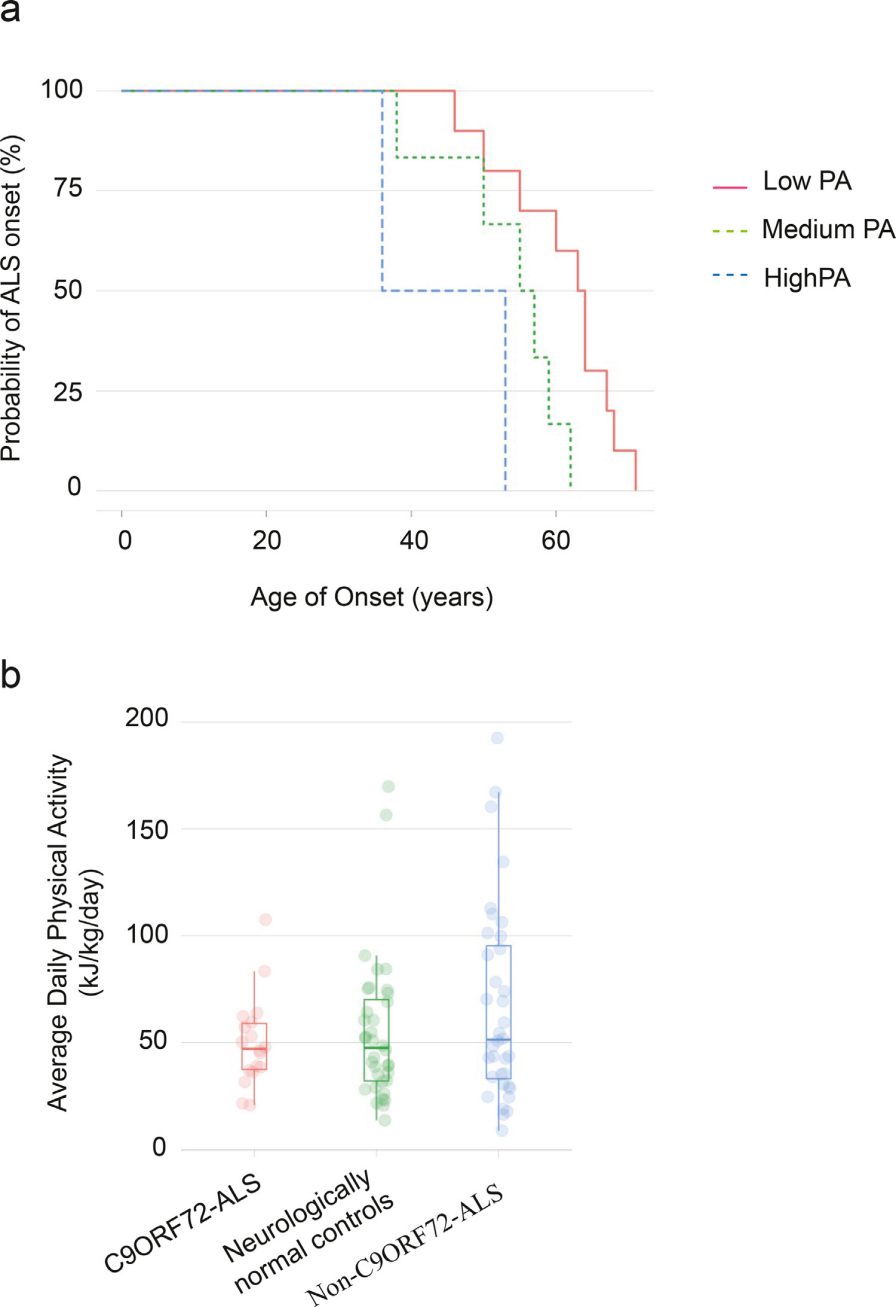
Case-control study of *C9ORF72*-ALS suggests a gene-environment interaction with physical activity (PA). **(a)** Historical PA measured by the validated HAPAQ questionnaire is inversely related to time to disease onset in C9ORF72-ALS. To aid interpretation curves were plotted after dividing historical PA into three interval-bins of equal size; low PA: 20.8-49.8kJ/kg/day; medium PA: 49.8-78.7kJ/kg/day; high PA: 78.7-108kJ/kg/day. **(b)** Measured historical PA is less variable in *C9ORF72*-ALS compared to non-*C9ORF72*-ALS patients and neurologically normal controls.

To investigate the possible gene-environment interaction further, we studied historical physical activity using the validated HAPAQ questionnaire [46] in a cohort of *C9ORF72-*ALS patients (n=17) compared to age and sex matched non-*C9ORF72*-ALS patients (n=34) and neurologically normal controls (n=34) (Table 4). We proposed that variable disease penetrance in carriers of a G4C2-repeat expansion of *C9ORF72* is in part due to differences in exercise history. In our model, an individual carrying a *C9ORF72* expansion is likely to develop ALS when they receive a certain ‘dose’ of exercise. Consistent with our model, age of ALS onset was negatively correlated with average daily physical activity in *C9ORF72*-ALS patients (Cox proportional hazards model, Wald test p=0.007; likelihood ratio test, p=0.01; concordance=74%; hazards ratio i.e. reduction in age of onset per 1kJ/kg/day change in exercise=1.03 years, with 95% confidence interval 1.009-1.06 years; Fig. 4a). The same was not true for non-*C9ORF72*-ALS patients (Cox proportional hazards model, Wald test p=0.1; likelihood ratio test, p=0.1; concordance=62%; hazards ratio=1.005, with 95% confidence interval 0.9987-1.012 years). After correcting for group size, variability in average physical activity was lower in *C9ORF72*-ALS compared to both non*-C9ORF72*-ALS (F-test, p=0.002; Fig. 4b) and neurologically normal controls (F-test, p=0.049; Fig. 4b). Reduced variability is consistent with our model because it suggests a homogeneous effect of physical activity in all *C9ORF72*-ALS patients. Our data do not exclude a role for physical activity in non-*C9ORF72*-ALS, but the increased heterogeneity in this group suggests that the effect size is variable.

**Table 4:** Clinical characteristics of *C9ORF72*-ALS cases and controls from case-control analysis.

	C9ORF72-ALS	Non-C9ORF72-ALS	Neurologically Normal Controls
Number in group	17	34	34
Proportion male (%)	58.8	58.8	52.9
Age (years)	56.4 (36-71)	57 (35-72)	57 (36-74)
ALS (%)	15 (88.2)	34 (100)	-
PMA (%)	2 (11.8)	0	-
PLS (%)	0	0	-
Limb onset (%)	10 (59)	21 (62)	-
Bulbar onset (%)	7 (41)	13 (38)	-

ALS = amyotrophic lateral sclerosis; PMA= progressive muscular atrophy variant; PLS= primary lateral sclerosis variant.

## 4 Discussion

ALS is an archetypal complex disease determined by interaction between genetic risk factors and environmental modifiers [2]. Consistent with this, the incidence of ALS varies between populations [52] and is lowest when ancestry is relatively mixed [53]. Understanding gene-environment interactions which increase the risk of ALS will lead to new personalised medicine approaches. For rare forms of ALS which are purely genetically determined, this is already a reality [54]. It is likely that the search for environmental risk factors has been hindered by studies which have failed to account for heterogeneity in both environmental exposures and ALS genetic subtypes: specific exposures are likely to be relevant only in the presence of specific genotypes. We have used two-sample MR to establish the basis for frequent and strenuous leisure-time exercise as a risk factor for ALS and we have also developed understanding of the specific genetic subtypes of ALS which may be responsible for this interaction.

The link between exercise and ALS risk has previously been controversial [8,9]. The advantage of a two-sample MR methodology is that it can avoid much of the bias and confounding factors which have hindered previous analyses. It should be noted that not all of our robust analyses were statistically significant however these tests are relatively underpowered [55], and our sensitivity analyses do not provide any evidence of significant instrument heterogeneity or directional pleiotropy to suggest that the IVW result is invalid.

Exercise is not one homogeneous exposure; in reality different types of exercise can impact different biological pathways and even different subtypes of motor neurons. Early in the disease process ALS is known to selectively affect motor neurons supplying type IIb fast twitch muscle fibres important for high intensity anaerobic exercise [16,17]. Consistent with this, our MR study does not support a causal role for low-intensity, infrequent exercise, but does support toxicity resulting from high-intensity, frequent, leisure-time exercise. The SSOE measure of exercise we utilised is negatively correlated with occupational activity suggesting that it is a specific measure of strenuous leisure-time activity [21]. Variations in the cardiovascular impact of occupational and leisure time activity are thought to represent more static occupational activity versus more dynamic leisure-time activity [19,56]. This suggests that intense leisure-time physical exercise is more likely to include strenuous anaerobic activity and might explain why this type of physical activity shows a strong association with ALS. Similarly our data show that static activity such as heavy DIY, is not associated with ALS.

Physical exercise is causally linked to several factors which may modify the risk of ALS including body fat percentage [50], educational attainment [11], cigarette smoking [57], type 2 diabetes mellitus [58], and head trauma [59], and each of these is a potential confounder; although we note that a recent MR study has disputed the causal relationship with smoking [60]. We are confident that the effect we observe is a direct effect of exercise and not an indirect effect via one or more of these factors. Firstly our MR study has demonstrated a dose-dependent relationship between SSOE and ALS risk [61]. It is difficult to explain this via an indirect effect which is more likely to be non-linear. Secondly our MR data do not support a causal relationship between body fat percentage and ALS suggesting that this is not in reality a potential confounder. Thirdly, increased physical exercise is associated with increased educational attainment [21], reduced cigarette smoking [62], and lower risk of type 2 diabetes [63], which should therefore reduce risk of ALS [11,57,58] in individuals who exercise more. This is the opposite direction of effect to that which we observed, and therefore these factors are unlikely to be a source of confounding. Finally, although risk of head trauma is linked to physical exercise, the SSOE measure we utilised gives equal weighting for heterogenous activities including some with minimal risk of head trauma (e.g. aerobics). We propose therefore that head trauma is unlikely to be a significant confounder of our data.

We discovered that ALS risk genes are differentially expressed following exercise and similarly, a significant number of exercise-induced biological pathways are enriched with ALS genetic risk. This provides a functional link between exercise and the activity of ALS risk genes. Many of these pathways have previously been linked to neurotoxicity, but our work places them upstream in the pathogenesis of exercise-induced ALS. Most prominently, genes linked to fibroblast growth factor (FGF) and nerve growth factor (NGF) signaling were highly enriched with ALS-associated rare variants. FGF1 is known to induce NGF expression in astrocytes and therefore these represent related pathways [64]. FGFs are highly expressed in motor neurons and FGF1 secretion can be stimulated by oxidative stress, hypoxia and serum starvation [64]. Produced by astrocytes, both NGF and FGF have been shown to cause motor neuron apoptosis under specific conditions *in vitro* and this signaling has been implicated in ALS pathophysiology [64,65]. A gap in our analysis is the link between gene expression changes in blood, and toxicity within the CNS. One possibility is that the two are linked by the mobilisation of energy stores to facilitate brain metabolism. NGF signaling has been implicated in glucose-stimulated insulin secretion [66] which is essential for CNS neurons. A recent ^18^F-FDG-PET study identified changes in brain metabolism induced by exercise which overlapped with changes previously observed in ALS [67]. Interestingly ALS-associated changes in brain metabolism were less extensive in patients with a significant exercise history, at the same stage of disease progression. The authors proposed that this may represent an exercise-induced failure of metabolic reserve leading to neurodegeneration.

We suggest that the pathways and genes we have highlighted, should be explored in detail to define a risk genotype for exercise-induced ALS. G4C2-repeat expansion of *C9ORF72* is the most common genetic risk factor for ALS, but the phenotype is variable and penetrance is incomplete suggesting a role for environmental modifiers [14]. We hypothesised that exercise may modify the penetrance of *C9ORF72*-expansions and precipitate *C9ORF72*-ALS as opposed to non-motor manifestations of *C9ORF72*-disease such as frontotemporal dementia (FTD). Consistent with this, we have shown that *C9ORF72* gene expression is down-regulated during exercise, which could act synergistically with haploinsufficiency caused by the G4C2-repeat expansion, so enhancing neurotoxicity [68,69]. Secondly we have shown that age of onset in *C9ORF72*-ALS is significantly correlated with the ‘dose’ of historical physical activity; a similar but weaker trend is present in non-*C9ORF72*-ALS patients, perhaps because of increased heterogeneity in this group. The fact that historical physical activity is more homogeneous in *C9ORF72*-ALS patients is consistent with our hypothesis that physical exercise has a modifier effect in this group. Finally, our data suggest that there may be a link between *C9ORF72* penetrance and exercise which could lead to lifestyle recommendations and potentially disease prevention. It is possible that individuals with a pathogenic *C9ORF72* expansion and a low dose of historical physical activity are not asymptomatic but may develop a different phenotype such as FTD. We were not able to assess this possibility because we selected for patients with a pure *C9ORF72*-ALS phenotype due to the difficulty in obtaining accurate historical physical activity in the context of significant cognitive impairment.

It is interesting that a recent MR study failed to identify a causal link between strenuous physical activity and sporadic ALS. This study considered only the type of exercise which had been performed in the previous four weeks, with no criteria for frequency or duration [11]. Similarly we did not detect a causal link between non-strenuous or infrequent physical activity and ALS. This observation of a relatively strict frequency cut-off could be linked to a threshold effect for penetrance; any measure of physical activity which included individuals exercising at a rate below the threshold would be significantly confounded and may mask an association. Crucially the SSOE measure used in the present MR study enabled us to capture only activity which was both frequent and intense.

In conclusion, the current evidence supports a complex causal relationship between physical exercise and ALS. However, it is clear that, for the majority of individuals, the health benefits of a physically active lifestyle markedly outweigh the risks. The key objective for future research is to understand which individuals are at risk of developing ALS if they exercise excessively and provide appropriate lifestyle counselling. Our work goes some way towards developing this aim and in particular, we propose that *C9ORF72* penetrance may be influenced by high levels of physical activity.

## Contributors

PJS, JCK and THJ conceived and designed the study. THJ, NG, ADFB, YCK, MPS, JCK and PJS were responsible for data acquisition. JCK, THJ, NG and PJS have verified the underlying data. THJ, NG, ADFB, YCK, MN, SZ, JCK and PJS were responsible for analysis of data. THJ, NG, ADFB, TM, CH, YCK, MN, SZ, MPS, JCK and PJS were responsible for interpretation of data. THJ, JCK and PJS prepared the manuscript with assistance from all authors. All authors meet the four ICMJE authorship criteria, and were responsible for revising the manuscript, approving the final version for publication, and for the accuracy and integrity of the work.

## Data sharing statement

Genome-wide association summary statistics are available and linked from the original manuscripts (**Methods**). Rare variant burden testing statistics are available through the Project MinE Data Browser at http://databrowser.projectmine.com/ [43]. Transcriptome changes in response to acute exercise are available from the original publication [44].

## Declaration of Competing Interest

M.P.S. is a cofounder of Personalis, Qbio, Sensomics, Filtricine, Mirvie and January. He is on the scientific advisory board of these companies and Genapsys. No other authors have competing interests.

## Appendix Supplementary materials

Image, application 1

Image, application 2
