# Supplementary material for: Physical exercise is a risk factor for amyotrophic lateral sclerosis: Convergent evidence from Mendelian randomisation, transcriptomics and risk genotypes
Source: eBioMedicine. 2021 May 26;68:103397. doi: 10.1016/j.ebiom.2021.103397 (PMC8170114; doi:10.1016/j.ebiom.2021.103397)
Supplement: Supplementary file 1 [file mmc1.pdf]

Supplementary Figure 1

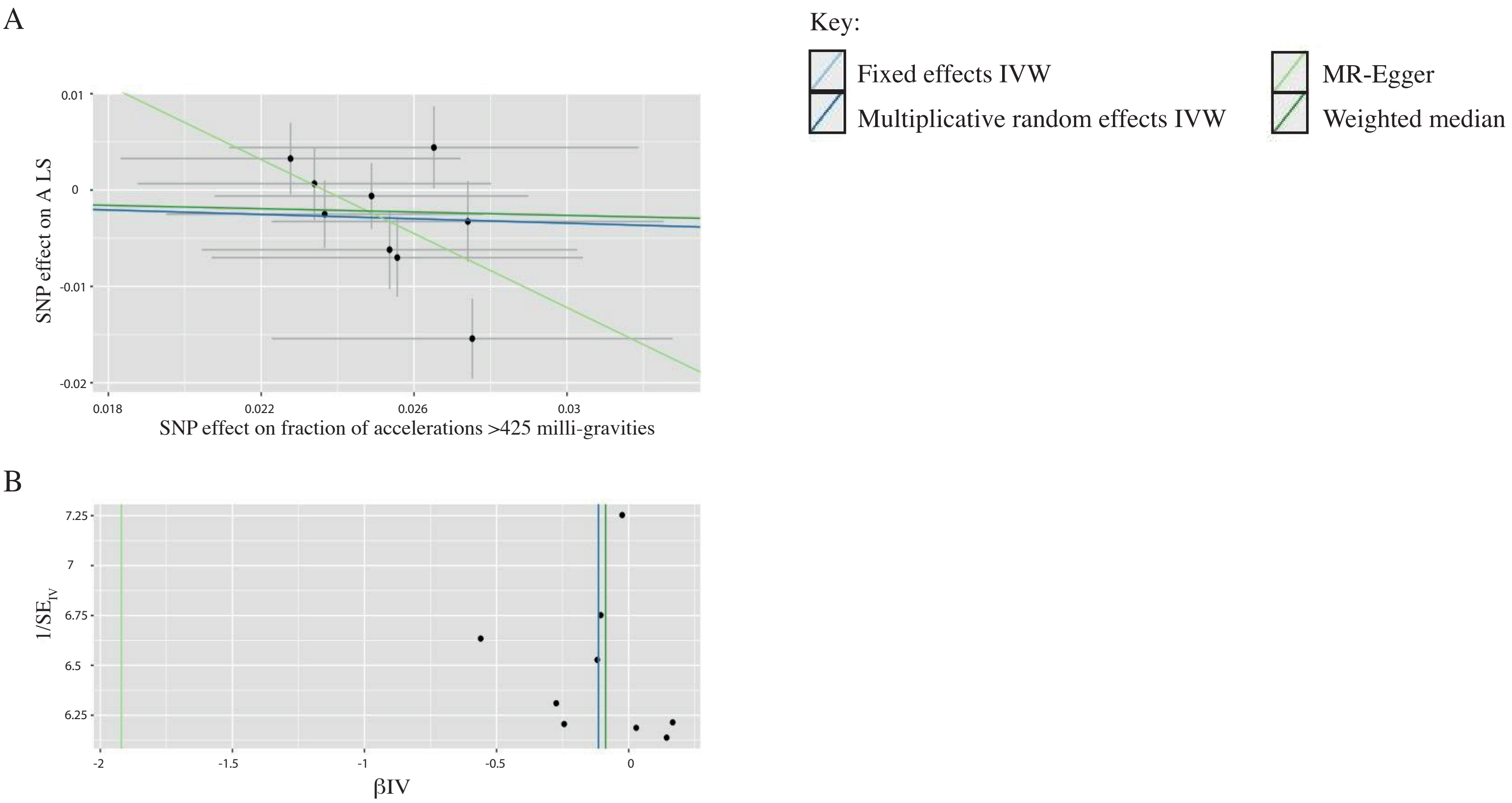

Supplementary Figure 2

A

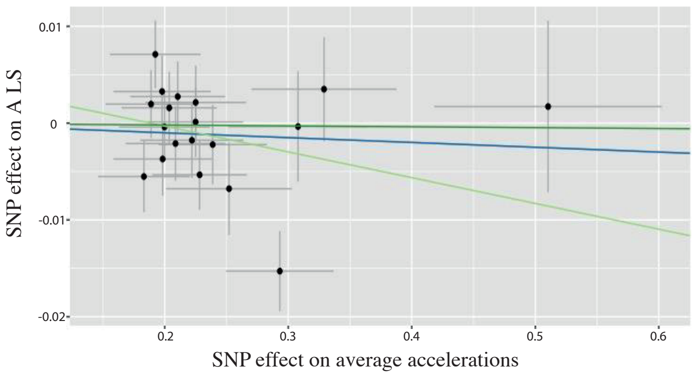

B

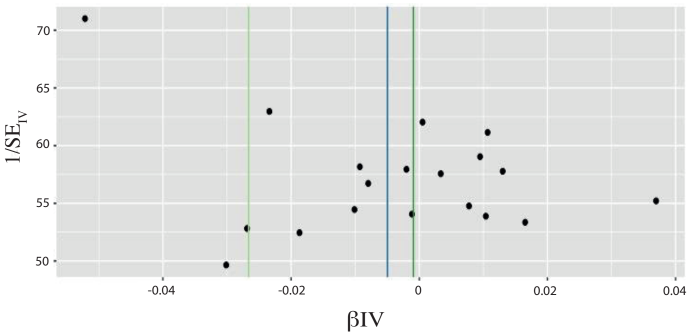

Key:

- Fixed effects IVW
- Multiplicative random effects IVW
- MR-Egger
- Weighted median

Supplementary Figure 3

A

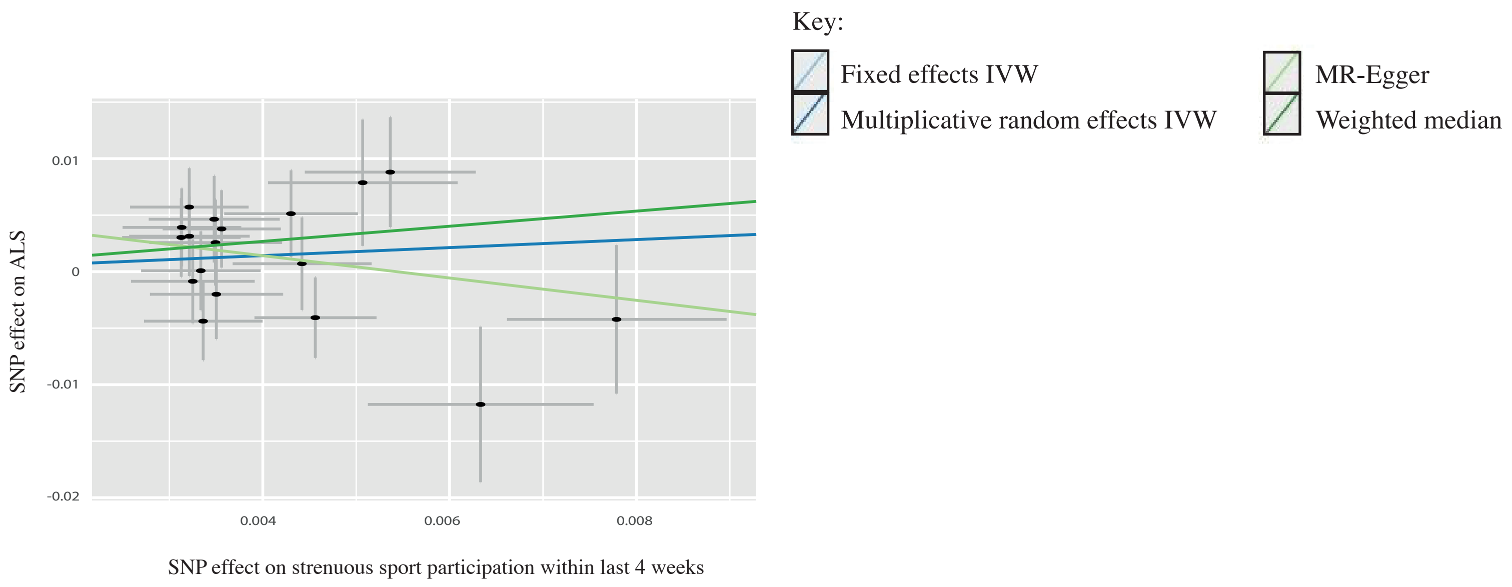

B

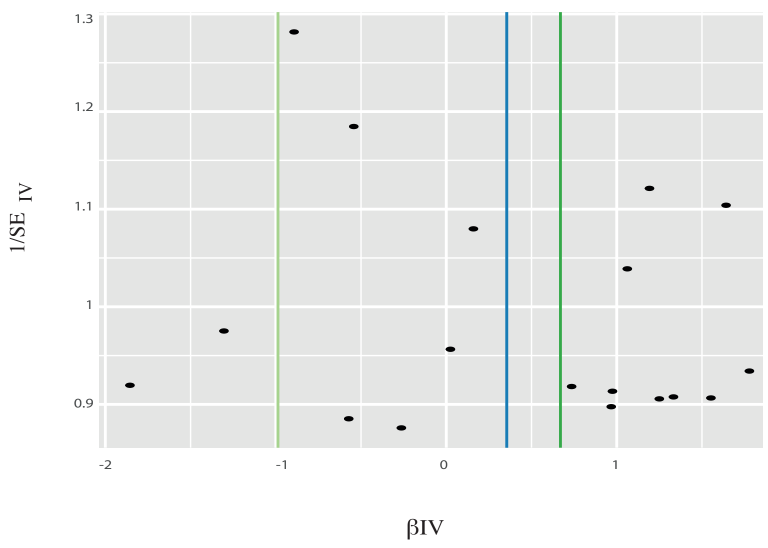

Supplementary Figure 4

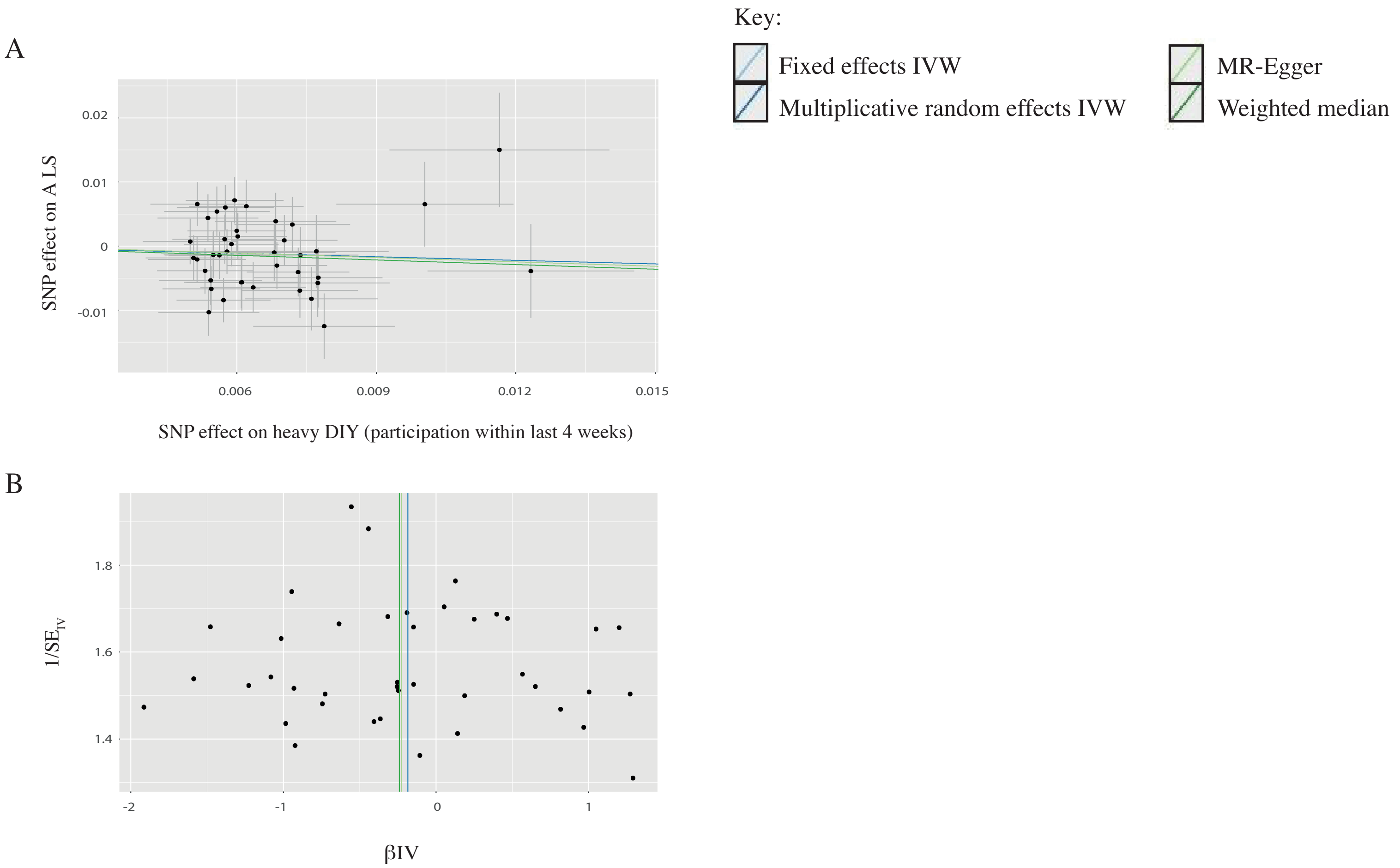

Supplementary Figure 5

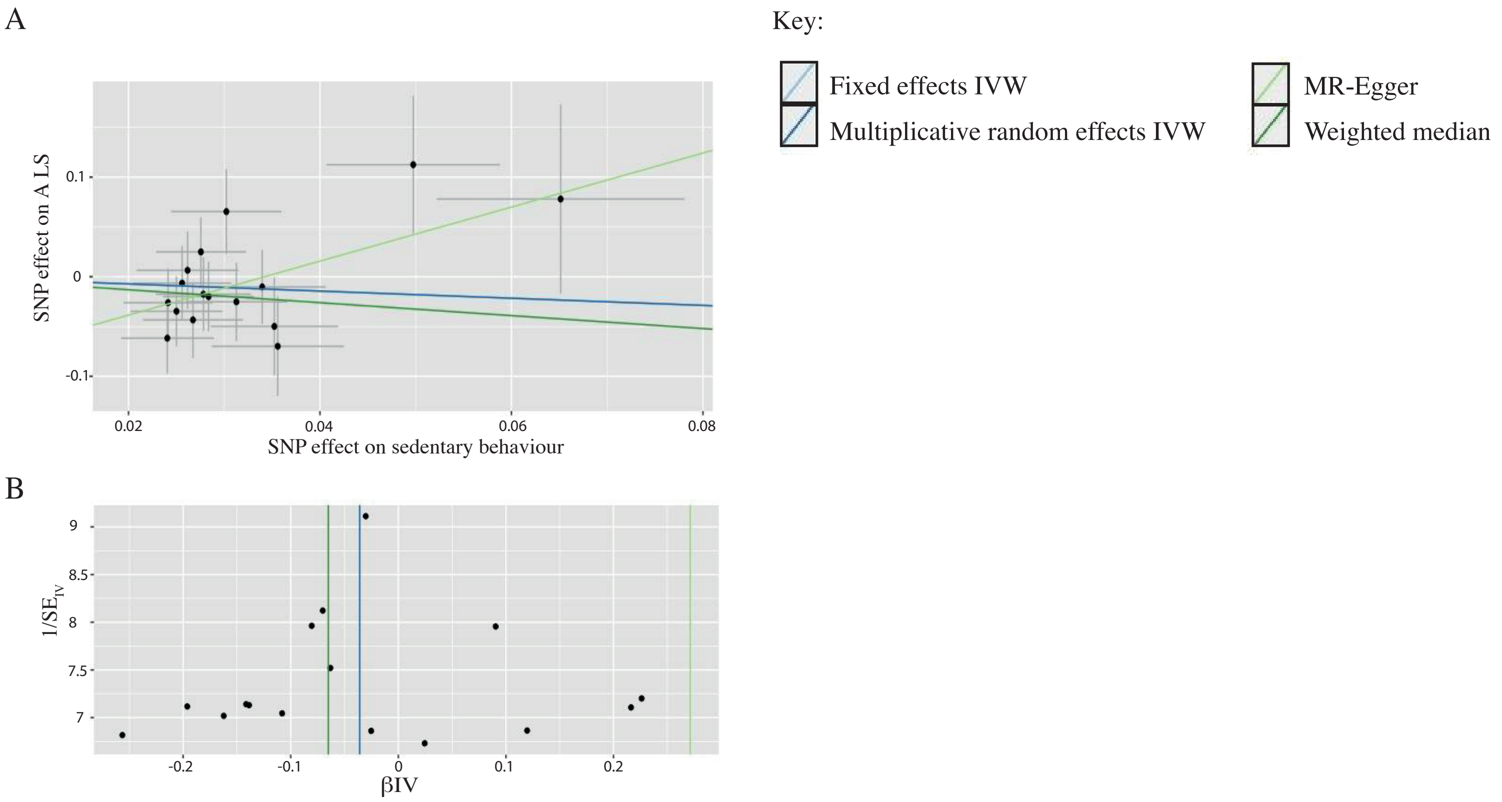

Supplementary Figure 6

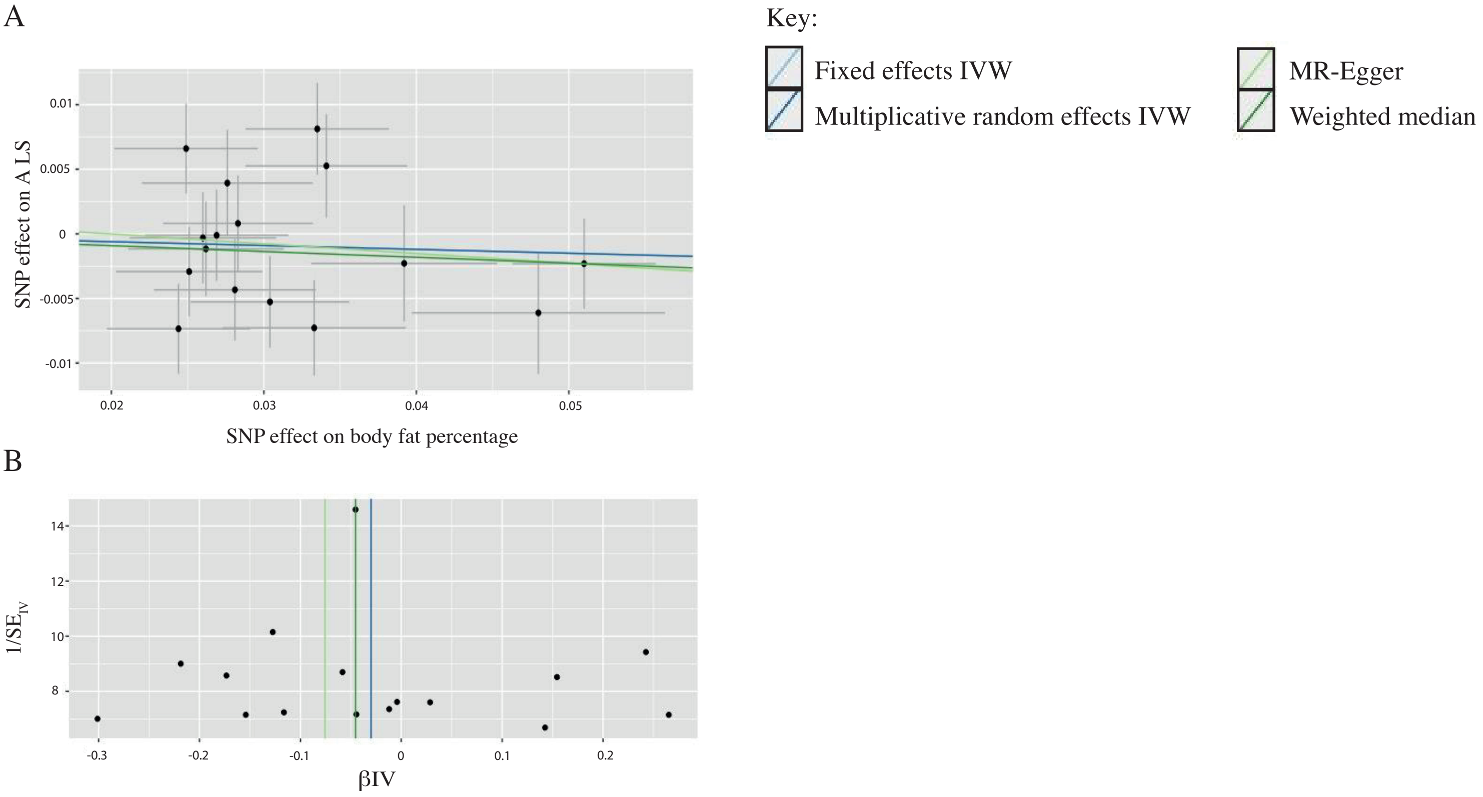

Supplementary Figure 7

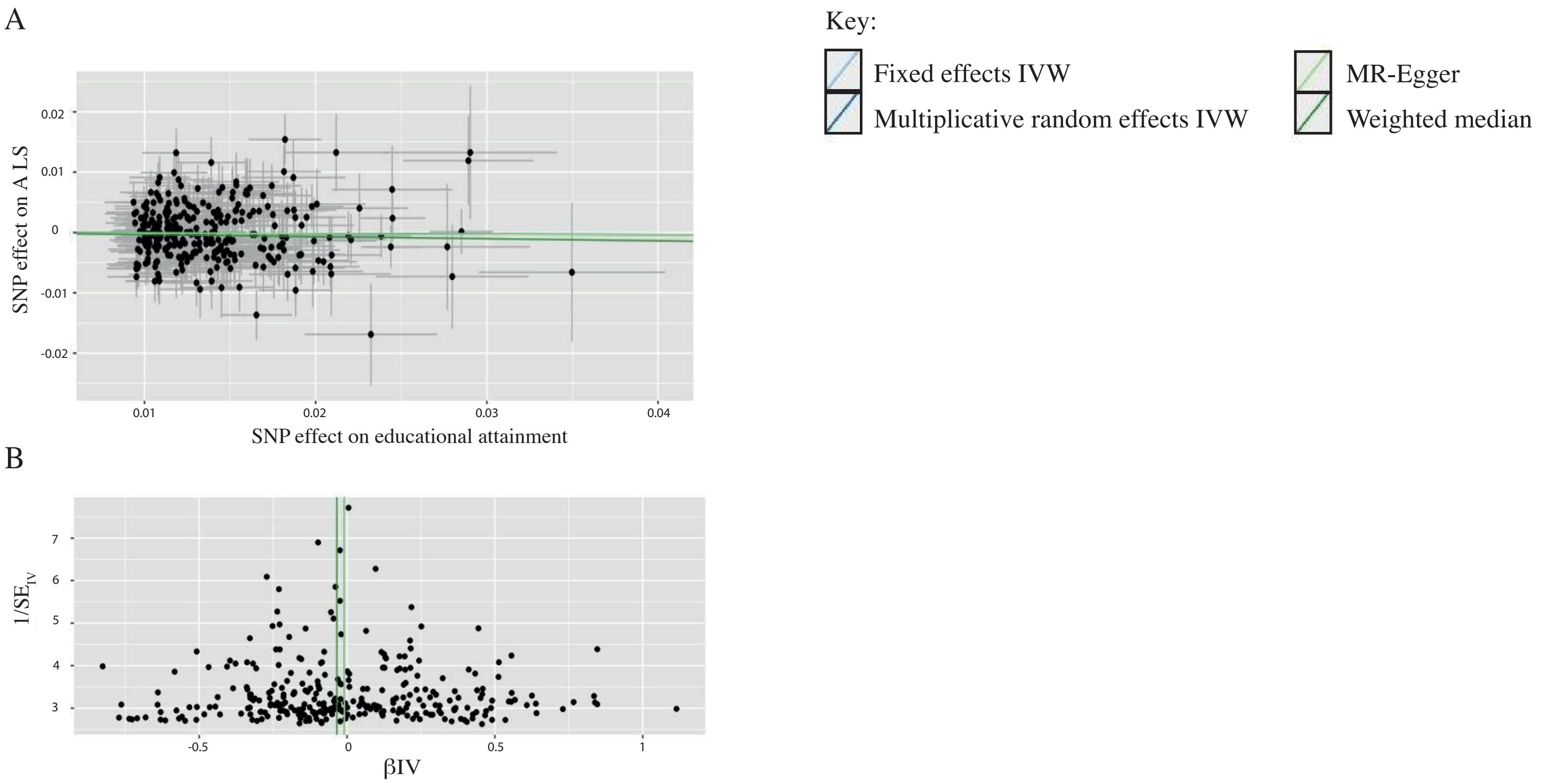

METHODOLOGICAL LIMITATIONS

Our study draws strength from the utilisation of several methodologies which provide convergent evidence that there is a causal relationship between strenuous leisure time PA and ALS. By combining multiple methods the impact of weakness in any given methodology on our overall conclusions is reduced.

As the effect of SSOE upon ALS has been modelled through the dichotomisation of a continuous variable, no particular significance can be appointed to the point of dichotomisation and a causal effect parameter cannot be meaningfully assigned.[1] We are therefore able to provide a positive direction of effect, but unable to assign an odds ratio to the exposure. Although the ten SNP conservative instruments for SSOE are positively correlated with ALS, it does not reach significance. This appears to be related to power and does not nullify our findings, but does increase the risk of pleiotropy having impacted upon our results, which we have minimised through several robust measures. Because individual SNPs explain a small fraction of complex traits such as those addressed in this study, a further potential source of MR error is insufficient power which may increase the risk of type 1 error in our low-intensity PA results. In order to address power limitations, we implemented a liberal exposure instrument in the MR analysis. The risk of type 1 error was reduced by calculation with a range of robust measures and assessments of instrument strength. Self-report questionnaire and interview data in this study is vulnerable to well recognised limitations including recall bias and social desirability bias though subjects were blinded to the study hypothesis.

R CODE UTILISED FOR MR AND BURDEN TESTING:

# MENDELIAN RANDOMISATION CODE UTILISED.
#This code was modified for each calculation by inputting the relevant exposure GWAS. The basic format is presented here.

# 1. Load required packages and load in exposure data locally

```
library(readr)
library(TwoSampleMR)
library(dplyr)

exposuregwas  <-  read_table2([GWAS EXPOSURE DATA LOCATION HERE,READR
FUNCTION MAY DIFFER ACCORDING TO THE GWAS FORMATTING])

renamedexposuregwas <- rename(exposuregwas , c(  [INSERT COLUMN NAME CHANGES IF
REQUIRED TO COMPLY WITH TwoSampleMR FORMAT]  ) )

dfEx <- as.data.frame(renamedexposuregwas)

sig_threshold<- dfEx %>% filter(pval < [INSERT NUMERICAL  SIGNIFICANCE LEVEL HERE] )

exposure <- format_data(sig_threshold , type = "exposure" )

exposure$exposure  <- gsub( "exposure" , “[INSERT EXPOSURE PHENOTYPE NAME HERE]”
, exposure$exposure )
```

# 2. Clump exposure data

```
clumpedexposure <- clump_data(exposure)
```

# 3. If you require a proxy SNP, insert now with this code (skip step if no proxy)

```
proxysnp <- dfEx %>%
  filter (SNP %in%  c(“[LIST OF PROXY SNPS GO HERE]”))

proxyexp <- format_data(proxysnp , "exposure")

proxyexp$exposure  <- gsub( "exposure" , “[INSERT EXPOSURE PHENOTYPE NAME HERE]”
, proxyexp$exposure )

clumpedexposure <- rbind(clumpedexposure, proxyexp)
```

# 4. Load outcome data locally

```
outcome_dat <- read_outcome_data(
  snps = clumpedexposure$SNP,
  [INSERT DETAILS OF OUTCOME DATA LOCATION AND COLUMN NAMES HERE AS PER
TwoSampleMR FORMAT]
)

outcome_dat$outcome  <- gsub( "outcome" , “[INSERT OUTCOME PHENOTYPE NAME
HERE]” , outcome_dat$outcome )
```

# 5. If you are uncertain of requirement for proxy SNPs , check if any SNPs are not present in both data sets (skip step if you’re certain no proxy is required)

```
missing <- clumpedexposure %>%
  filter ( !SNP %in% outcome_dat$SNP)

View(missing)
```

# 6. If an SNP was identified to be missing in step 5, you need to locate an appropriate proxy. If no proxy is required skip this step. Note the first column in the data frame must be the SNP column.

```
library(LDlinkR)
for (i in 1:nrow(missing))  {
  x <- LDproxy(missing [i , 1], pop = "EUR", r2d = "r2", token = “[INSERT TOKEN ID HERE]”)

  eligible <- x[x$R2 >= 0.9 , ]

  A <- dfEx %>%
    filter ( SNP %in% eligible$RS_Number)

  Common <- [DATA FRAME CONTAINING THE ENTIRE OUTCOME GWAS HERE] %>%
    filter ( SNP %in% A$SNP)

  R2vals <- eligible %>%
    filter( RS_Number %in% Common$SNP)
  missing$proxySNP[i] <- R2vals[1 , 1]
}

View(missing) #missing now contains a column of proxy SNPs to use at step 3
```

# 7. If you have used a proxy SNP, there will be two different 'id.exposure' names, they must be changed to the same value for harmonisation (if not proxy was inserted then skip this step)

```
clumpedexposure$id.exposure  <- gsub( "[ID 1]" , "[ID 2]" , clumpedexposure$id.exposure )
```

# 8 Harmonise the data, the default setting is that palindromic alleles with intermediate effect allele frequency are removed

```
dat <- harmonise_data(
  exposure_dat = clumpedexposure,
  outcome_dat = outcome_dat ,
)
```

# 9. Add a column of PVE and F statistics. During the above processes only EAF have been retained and therefore MAF needs to be calculated during this process.

# A) Calculate a per-SNP F statistic

```
dat$EAF2 <- (1 - dat$eaf.exposure)
dat$MAF <- pmin(dat$eaf.exposure, dat$EAF2)
PVEfx <- function(BETA, MAF, SE, N){
  pve <- (2*(BETA^2)*MAF*(1 - MAF))/
    ((2*(BETA^2)*MAF*(1 - MAF)) + ((SE^2)*2*N*MAF*(1 - MAF)))
  return(pve)
}
dat$PVE <- mapply(PVEfx,
                  dat$beta.exposure,
                  dat$MAF,
                  dat$se.exposure,
                  N = [INSERT EXPOSURE GWAS POPULATION SIZE)
dat$FSTAT <- (([INSERT EXPOSURE GWAS POPULATION SIZE] - 1 - 1)/1)*(dat$PVE/(1 -
dat$PVE))
```

# B) Calculate a total instrument F statistic

```
(([INSERT EXPOSURE GWAS POPULATION SIZE] - [INSERT TOTAL NUMBER OF SNPS IN
INSTRUMENT] - 1)/[INSERT TOTAL NUMBER OF SNPS IN INSTRUMENT])*([INSERT TOTAL
PVE]/(1 - [INSERT TOTAL PVE]))
```

#10. Calculate the cochran’s Q, egger intercept, causal estimates, I^2, MR-PRESSO , leave one out and develop graphs

# Cochran's Q

```
View(mr_heterogeneity(dat))
```

#Egger intercept

```
View(mr_pleiotropy_test(dat))
```

#I^2

```
Isq( dat$beta.exposure , dat$se.exposure)
```

#MR-PRESSO

```
run_mr_presso(dat)
```

#MR causal estimates and odds ratios (OR not used in this paper due to binary exposure)

```
View(generate_odds_ratios(  mr(dat , method_list =  c("mr_ivw_fe" , "mr_ivw_mre" ,
"mr_egger_regression" , "mr_weighted_median"))))
```

#Leave one out analysis

```
View(mr_leaveoneout(dat, method = [INSERT MOST APPROPRIATE MR METHOD AS PER
TwoSampleMR FORMAT]))
```

#Scatter plot

```
mr_scatter_plot(mr(dat, method_list = c( "mr_ivw_fe" , "mr_ivw_mre" , "mr_egger_regression" ,
"mr_weighted_median")), dat)
```

#Funnel plot

```
mr_funnel_plot( mr_singlesnp(
  dat,
  parameters = default_parameters(),
  single_method = "mr_wald_ratio",
  all_method = c("mr_ivw_fe" , "mr_ivw_mre" , "mr_egger_regression" , "mr_weighted_median")
))
```

#BURDEN TESTING CODE UTILISED FOR EXERCISE-RELATED GENES IN ALS PATIENTS.
#This has not been made generic, as only one set of data was analysed with this code (i.e. the exercise transcript and ALS transcript data). The data utilised is available in supplementary table 2.5 in the study of origin. We only imported pathways associated at 2 minutes post-exercise into R.

# Pre analysis, load in relevant packages

```
library(dplyr)


# 1. Load in the transcripts which are differentially expressed in response to exercise
from Contrepois et al. 2020.
# split each pathway into its constituent genes.
# name according to the pathway.

transcript_upregulated_exercise <- read_csv("transcript_upregulated_exercise.csv")

two_mins <- transcript_upregulated_exercise [ , c("Pathway" , "sig_genes_2 min" ) ]

split <- str_split_fixed(two_mins$`sig_genes_2 min` , "[,/]" , 180 )

row.names (split) <- two_mins$`Pathway`

# 2. Load in the Project MinE transcript data  (MAF1%)

ProjectMinE_Transcripts_0_01_results <-
read_table2("burden/ProjectMinE.Transcripts.0.01.results.txt")

# 3. identify which genes enriched in exercise are significantly related to MND.

project_mine_enriched <- subset(ProjectMinE_Transcripts_0_01_results , GeneName %in%
split ,)

p_missense <- project_mine_enriched %>%
  group_by(GeneName) %>%
  slice(which.min(p.firth.dis.dam.miss))

sigmiss <- p_missense[p_missense$p.firth.dis.dam.miss <0.05, ]

# 4. produce a vector of significant gene names

genes_sig <- sigmiss$GeneName[!duplicated(sigmiss$GeneName)]

# 5. Create a loop which calculates the proportion of exercise-related genes in each
pathway which are significant in MND.
# Note, 1 is subtracted from the length as a blank box is counted at the end of each row.
result <- rep(0,length (split[,1]))

names(result) <- row.names (split)
```

```
for (i in 1:nrow(split))
{
  x1 <- unique (split[i,])
  x2 <- which (genes_sig %in% x1)
  result[i] <- (length (x2) / (length (x1)-1))
}
```

# 6. Create a loop which calculates the total number of genes in each pathway. 1 is subtracted from the total genes to remove empty cells as a count.

```
Total_Genes <- rep(0 , nrow(split))

for (i in 1:nrow(split))
{
  x1 <- unique (split[i,])

  Total_Genes[i] <- (length (x1)-1)
}
```

# 7. Create a data frame which contains the pathway and gives information about the total number of significant genes.

```
result.df <- data.frame( result , two_mins , Total_Genes)

View(result.df)
```

# 8. Compare the results to 5000 random iterations

```
genes_only <- ProjectMinE_Transcripts_0_01_results %>%  distinct(GeneName)

gene_sig <- sigmiss %>% distinct(GeneName)

results <-matrix (nrow = 323 , ncol=5000 )

for ( x in 1:5000) {
  for (i in 1:323)
  {
    a <- data.frame(i , result.df[ i , 2] , c(sample_n(genes_only , result.df[i , 4], replace = FALSE)))
    b <- which(a$GeneName %in% gene_sig$GeneName)
    c <- length(b) / result.df [i,4]
    d <- c >  result.df[i , 1]
    results [i , x ] <- d
  }
}
```

```
pvals <- matrix(nrow = 323 , ncol =1)
for ( m in 1:323)
{
  A <- sum(results[ m , ])
  B <- A / 5000
  pvals [m , 1] <- B
}

table <- cbind(result.df[ 1:nrow(results), 2] , result.df[1:nrow(results), 4], pvals)
```

# 9. Calculate the false discovery rate . Note, prior to this stage we calculated p values for very significant pathways by operating 30,000 random iterations as per the methods section. Therefore, the table was edited with new p values for very significant pathways before this stage.

```
FDR <- p.adjust(table[ , 3] , method = "BH", n = length(table))

fulltable <- cbind(table , FDR )

write.csv(fulltable,file = file.choose(new = T))
```

1. REFERENCE:

1 Burgess S, Labrecque JA. Mendelian randomization with a binary exposure variable: interpretation and presentation of causal estimates. European Journal of Epidemiology. 2018; **33**: 947–52.
